# Salivary Matrix Metalloproteinase-8 and -9 and Myeloperoxidase in Relation to Coronary Heart and Periodontal Diseases: A Subgroup Report from the PAROKRANK Study (Periodontitis and Its Relation to Coronary Artery Disease)

**DOI:** 10.1371/journal.pone.0126370

**Published:** 2015-07-01

**Authors:** Nilminie Rathnayake, Anders Gustafsson, Anna Norhammar, Barbro Kjellström, Björn Klinge, Lars Rydén, Taina Tervahartiala, Timo Sorsa, PAROKRANK Steering Group

**Affiliations:** 1 Karolinska Institutet, Department of Dental Medicine, Division of Periodontology, Stockholm, Sweden; 2 Karolinska Institutet, Karolinska University Hospital Solna, Department of Medicine, Cardiology Unit, Stockholm, Sweden; 3 University of Helsinki, Helsinki University Central Hospital, Department of Oral and Maxillofacial Diseases, Helsinki, Finland; 4 University of Helsinki, Helsinki University Central Hospital, Department of Periodontology, Helsinki, Finland; 5 Department of Periodontology, Faculty of Odontology, Malmo University, Malmo, Sweden; National Center for Scientific Research Demokritos, GREECE

## Abstract

**Background and Objective:**

Matrix metalloproteinase (MMP) -8, -9 and myeloperoxidase (MPO) are inflammatory mediators. The potential associations between MMP-8, -9, MPO and their abilities to reflect cardiovascular risk remains to be evaluated in saliva. The objective of this study was to investigate the levels and associations of salivary MMP-8, -9, MPO and tissue inhibitors of metalloproteinase (TIMP)-1 in myocardial infarction (MI) patients and controls with or without periodontitis.

**Materials and Methods:**

200 patients with a first MI admitted to coronary care units in Sweden from May 2010 to December 2011 and 200 controls matched for age, gender, residential area and without previous MI were included. Dental examination and saliva sample collection was performed 6-10 weeks after the MI in patients and at baseline in controls. The biomarkers MMP -8, -9, MPO and TIMP-1 were analyzed by time-resolved immunofluorescence assay (IFMA), Western blot and Enzyme-Linked ImmunoSorbent Assay (ELISA).

**Results:**

After compensation for gingivitis, gingival pockets and smoking, the mean salivary levels of MMP-8 (543 vs 440 ng/mL, p = 0.003) and MPO (1899 vs 1637 ng/mL, p = 0.02) were higher in non-MI subjects compared to MI patients. MMP-8, -9 and MPO correlated positively with clinical signs of gingival/periodontal inflammation while TIMP-1 correlated mainly negatively with these signs. The levels of latent and active forms of MMP-8 did not differ between the MI and non-MI groups. Additionally, MMP-8, MPO levels and MMP-8/TIMP-1 ratio were significantly higher in men compared to women with MI.

**Conclusions:**

This study shows that salivary levels of the analyzed biomarkers are associated with periodontal status. However, these biomarkers could not differentiate between patients with or without a MI. These findings illustrate the importance to consider the influence of oral conditions when analyzing levels of inflammatory salivary biomarkers.

## Introduction

Periodontal disease is an inflammatory condition appearing commonly in the adult population [1]. The prevalence of periodontal diseases varies, from gingivitis (prevalence 90%) to moderate (35%) and severe (5–8%) periodontitis [2]. An association between periodontitis and cardiovascular disease (CVD) is known but the pathological mechanisms and potential links between the two diseases are not yet completely clarified [3].

Matrix metalloproteinases (MMP) are calcium- dependent zinc containing endopeptidases that play an important role in normal physiological processes such as tissue development and remodeling as well as in pathological processes [4]. Twenty-three genetically distinct MMPs have been identified in humans. They have an anti-inflammatory (defensive) as well as a pathogenic (tissue destructive) role and are involved in the pathogenesis of large number of different diseases and conditions [5]. MMPs are produced in latent, non-active pro-forms and activated extra- or intracellularly depending upon the structure of the MMP molecule [6]. The main inhibitors of MMPs are tissue inhibitors of metalloproteinases (TIMPs) that restrict extracellular matrix component breakdown [7].

MMP-8, also known as collagenase-2 or neutrophil collagenase, is related to inflammatory conditions. It is expressed mainly by neutrophils [8] but also by endothelial and smooth muscle cells and macrophages in atherosclerotic lesions [9, 10]. Apoptosis of endothelial cells and release of MMP-8 promote the conversion of stable lesions to unstable lesions and lead to plaque rupture [11] and elevated MMP-8 levels have been found in rupture prone and vulnerable plaques [10]. Furthermore, salivary MMP-8 levels are associated with progressive loss of attachment in periodontitis [8, 12]. MMP-9 (gelatinase B) is increased in stimulated whole saliva from inflamed periodontal sites [13, 14]. Increased levels of MMP-9 in serum have also been shown to be a marker of cardiovascular disease [15].

Myeloperoxidase (MPO) is strongly associated with on-going inflammation [16] but has widely been used as a marker of both acute and chronic inflammatory conditions. Its main role is to generate hypochlorous acid to kill bacteria. The oxidative function of MPO also activates latent forms of proMMP-8 and -9 and inactivates TIMPs [17, 18]. MPO is associated with CVD and elevated salivary MPO levels have been demonstrated in periodontal disease [19–22].

The objective of the current study was to investigate salivary MMP-8, -9, TIMP-1 and MPO levels in relation to MI and periodontal disease.

## Materials and Methods

### Ethics Statement

The case-control PAROKRANK study (Periodontal disease and the relation to myocardial infarction), including the study protocols for participant recruitment, and informed consent for participants, were approved by the regional ethical review board (Dnr. 2008/152-31/2) Karolinska Institutet, Stockholm, Sweden. All participants gave their written informed consent.

### Study population

Two-hundred patients, < 75 years old and admitted to a coronary care unit with a first MI were enrolled. Enrollment for this sub-study occurred between May 2010 and December 2011 at eleven participating hospitals in Sweden. The MI was diagnosed according to international definitions [23, 24]. In addition, 200 control subjects with no history of MI and matched for age, gender and postal code were recruited from the national population registry. Study participants were excluded if they had undergone cardiac valvular surgery or had language barriers preventing them to complete study procedures.

### Study protocol

Study participants attended the local cardiology and dental departments for clinical and laboratory measurements, questionnaire evaluation and for extensive periodontal examination including salivary collection. Patients were examined 6–10 weeks after the MI and the matched controls in close proximity thereafter.

#### Dental examination and evaluation

All participants fasted one hour prior to the dental examination. Saliva and bacterial samples were collected first and the subsequent dental examination comprised number of present teeth, soft tissue pathologies, dental caries and periodontal status, including probing pocket depth (PPD) at four sites per tooth, bleeding on probing (BOP) and present or absent plaque score. Mobility and furcation involvement were also recorded.

#### Collection and preparation of salivary samples

Sample collection: Stimulated saliva samples were obtained by chewing paraffin wax up to 10 minutes. The produced saliva was collected into a graded test-tube. The saliva collection continued until 2 mL of saliva was obtained or until 10 minutes had passed. The collected amount was determined, excluding the foam. Collected samples were immediately frozen at -20°C or lower until processing. Each vial was thawed and centrifuged at 500g for 5 minutes, at 5°C. The supernatants were aliquoted into 1.5 mL Eppendorf tubes (Eppendorf, Hauppauge, NY, USA), and stored at -80°C. Each saliva aliquot was used twice for the determination of selected biomarkers.

### Determination of biomarker levels in saliva

The MMP-8 concentrations were determined by an immunofluorescence assay (IFMA). The monoclonal MMP-8 specific antibodies 8708 and 8706 (Medix Biochemica, Kauniainen, Finland) were used as a catching antibody and a tracer antibody, respectively. The tracer antibody was labeled using europium-chelate [25]. The assay buffer contained 20 mM Tris-HCl, pH 7.5, 0.5 M NaCl, 5 mM CaCl_2_, 50 μM ZnCl_2_, 0.5% BSA, 0.05% sodium azide and 20 mg/L diethylenetriaminepentaacetic acid (DTPA). Samples were diluted in assay buffer and incubated for one hour, followed by incubation for one hour with tracer antibody. Enhancement solution was added and after 5 min fluorescence was measured using a 1234 Delfia Research Fluorometer (Wallac, Turku, Finland). The specificity of the monoclonal antibodies against MMP-8 corresponded to that of polyclonal MMP-8. The interassay coefficient of variation (CV) % was 7.3%. The detection limit for the assay is 0.08 ng/mL. Salivary MMP-9, TIMP-1 and MPO levels were determined using ELISA kits according to the manufacturer’s instructions (R&D Systems, Minneapolis, MN, USA; Amersham Biosciences, Buckinghamshire, UK; Immundiagnostik, Bensheim, Germany, respectlively) [26]. Furthermore, salivary samples were examined by Western blotting using polyclonal antibodies against MMP-8. The immunoblot was quantitated by densitometric computer scanning, and the data expressed as arbitrary units [27].

### Statistical analysis

Statistical analyses were performed using Statistical Package for Social Sciences (SPSS) version 22 (SPSS Inc., Chicago, IL). Differences were considered significant at a probability level of p < 0.05. To compare the ratio of categorical variables Chi-squared test was used. The amounts of the analyzed biomarkers are expressed as median and interquartile range and the significance of the differences calculated with Mann-Whitney U test. However, to allow compensation for smoking, gender and periodontal pockets, when comparing biomarker levels in patients with and without MI, a General Linear Model test was performed and the values are expressed as mean ± SD. The resulting p-values were verified with a logarithmic transformation of the data. Correlations were calculated by using Spearman´s correlation tests.

## Results

The results are based on examination of patients 6–10 weeks after the MI and the matched controls at baseline. The mean age was 61±8 in both groups and 84% were male. Tobacco use or incidence of hypertension and diabetes did not differ between the study groups. Only the total pathogenic periodontal pocket depth differed between the groups, median 48 mm in the MI group compared to 38 mm in the non-MI group (p < 0.01) (Table 1).

**Table 1:** Characteristics of the study population.

Condition/Variable	MI (n = 200)	non-MI (n = 200)	*p*
Male gender; n (%)	168 (84)	168 (84)	1.00
Age (Mean ± SD)	61±8	61±8	0.73
**Smoking**; n (%)			
Yes	19 (10)	26 (13)	0.34
No	74 (38)	83 (42)	
Ex-smoker	103 (52)	91 (45)	
**Snuffing**; n (%)			
Yes	10 (5)	24 (12)	0.07
No	158 (81)	152 (76)	
Ex-snuffer	25 (13)	23 (12)	
Hypertension; n (%)	83 (42)	67 (34)	0.20
Diabetes; n (%)	18 (9)	10 (5)	0.28
**Medical treatment**; n (%)			
ACEI	136 (68)	21 (11)	0.001
Aspirin	194 (97)	26 (13)	0.001
Beta blocker	180 (90)	31 (16)	0.001
Statins	188 (94)	37 (19)	0.001
Anti-inflammatory drugs	4 (2)	10 (5)	0.10
**Clinical periodontal status**: Median (IQR)			
Plaque	53 (50)	45 (47)	0.34
BOP	26 (39)	21 (34)	0.08
PPD 4–5 mm	11 (18)	9 (17)	0.12
PPD ≥ 6 mm	0 (2)	1 (0)	0.08
Total PPD mm	48 (88)	38 (71)	0.01

ACEI = angiotensin-converting enzyme inhibitors, IQR = Interquartile range, BOP = bleeding on probing, PPD = probing pocket depth. Significance of the differences calculated with Students t-test (age), Chi 2 or Mann-Whitney´s U test.

The mean salivary levels of MMP-8 and MPO were significantly higher in non-MI subjects after adjustment for smoking, BOP, PPD 4–5 mm and ≥ 6 mm (MMP-8; 543 vs 440 ng/mL, p = 0.003) and MPO; 1899 vs 1637 ng/mL, p = 0.02), (Table 2). In addition, the different enzyme forms (pro- and active) of MMP-8 obtained as densitometric units from scanning Western blot images did not differ between MI and non-MI patients. Active MMP-8 levels were higher in the non-MI group but it didn’t reach the significant level (7.12 (16.6) vs 9.12 (18.1), p = 0.06) (Table 3).

**Table 2:** The mean levels of MMP-8, -9, MPO, TIMP-1 and the ratios of MMP-8 and -9/TIMP-1 in stimulated saliva from MI and non-MI subjects.

Condition/Biomarker	MI (n = 200)	non-MI (n = 200)		
	Mean ± SD	Mean ± SD	*p*1	*p*2
MMP-8 (ng/mL)	440 ± 377	543 ± 398	0.008	0.003
MMP-9 (ng/mL)	260 ± 257	264 ± 217	0.88	0.78
MPO (ng/mL)	1637 ± 1386	1899 ± 1447	0.06	0.02
TIMP-1 (ng/mL)	208 ± 119	229 ± 129	0.08	0.09
MMP-8/ TIMP-1	1.63 ± 2.79	1.62 ± 2.48	0.99	0.60
MMP-9/ TIMP-1	0.74 ± 1.45	0.56 ± 0.81	0.12	0.20

*p*1 indicates significance of the differences after a bivariate comparison. *p*2 indicates the significance after compensation for differences in smoking, BOP, PPD 4–5 mm and PPD ≥ 6 mm.

**Table 3:** Median and interquartile range (IQR) of pro- and active forms of MMP-8 between MI patients and non-MI subjects.

Condition/Enzyme form	MI	Non-MI	
	Median (IQR)	Median (IQR)	*p*
Pro MMP-8 (au)	7.63 (6.6)	8.19 (6.3)	0.86
Active MMP-8 (au)	7.12 (16.6)	9.12 (18.1)	0.06

au = arbitrary unit.

The correlations of salivary MMP-8, -9, MPO, TIMP-1 are shown in Table 4. Most of the analyzed biomarkers correlated significantly with the other biomarkers. TIMP-1 showed a negative correlation with MMP-8, -9 and MPO levels.

**Table 4:** Correlations (r) of salivary MMP-8, -9, MPO, TIMP-1.

Biomarkers	MI (n = 200)			Non-MI (n = 200)		
	MMP–8	MPO	TIMP–1	MMP–8	MPO	TIMP–1
MMP-9	0.5**	0.4**	-0.3**	0.3**	0.2**	-0.1
MMP-8		0.7**	-0.2**		0.7**	-0.1
MPO			-0.1			-0.1

** Correlation is significant at the 0.01 level.

A weak but significant positive correlation between most of the analyzed biomarkers and the included clinical variables was seen in both study groups (Table 5). There was a negative correlation between TIMP-1 and clinical variables.

**Table 5:** Correlations (r) of salivary MMP-8, -9, MPO, TIMP-1 and the ratios of MMP-8 and -9/TIMP-1 measurements and periodontal parameters in MI (n = 200) and non-MI (n = 200) subjects.

Clinical variable/ Biomarker	Plaque		BOP		PPD 4–5 mm		PPD ≥ 6mm		Total PPD mm	
	MI	non-MI	MI	non-MI	MI	non-MI	MI	non-MI	MI	non-MI
MMP-8	0.13	0.24**	0.30**	0.37**	0.14	0.17*	0.20**	0.17*	0.01	0.09
MMP-9	0.03	0.07	0.25**	0.08	0.22**	0.01	0.29**	0.02	0.18*	-0.05
MPO	0.07	0.22**	0.30**	0.36**	0.16*	0.22*	0.16*	0.19**	0.05	0.16*
TIMP-1	-0.18*	-0.38**	-0.16*	-0.22**	-0.10	-0.01	-0.14	-0.10	-0.08	-0.01
MMP-8/ TIMP-1	0.19**	0.39**	0.34**	0.43**	0.17*	0.13*	0.23**	0.15*	0.06	0.05
MMP-9/ TIMP-1	0.11	0.27**	0.27**	0.20**	0.22**	-0.04	0.29**	0.05	0.18*	-0.08

BOP = bleeding on probing, PPD = probing pocket depth,

* Correlation is significant at the 0.05 level;

** Correlation is significant at the 0.01 level

The association between biomarkers and gender showed that MMP-8, MPO and MMP-8/TIMP-1 ratio were significantly higher in men with MI. MMP-8 levels in men without MI were also significantly higher compared to women without MI. There were no differences between smoker and non-smokers (Table 6).

**Table 6:** Biomarkers related to smoking and gender of MI patients and non-MI subjects.

Biomarker	MI (n = 200)						non MI (n = 200)					
	Smoking			Gender			Smoking			Gender		
	Median (IQR)			Median (IQR)			Median (IQR)			Median (IQR)		
	Current smokers	Non-smokers	*p*	Women	Men	*p*	Current smokers	Non-smokers	*p*	Women	Men	*p*
MMP-8	350 (587)	275 (536)	0.23	182 (255)	388 (579)	0.01	463 (602)	478 (739)	0.62	335 (598)	487 (633)	0.02
MMP-9	233 (235)	151 (247)	0.09	144 (256)	189 (246)	0.31	240 (277)	225 (260)	0.33	203 (325)	245 (257)	0.19
MPO	1372 (1747)	1077 (1168)	0.16	693 (1102)	1340 (1587)	0.01	1526 (1433)	1454 (1623)	0.99	1181 (1759)	1535 (1593)	0.07
TIMP-1	191 (127)	171 (114)	0.15	180 (130)	187 (125)	0.39	215 (174)	197 (150)	0.31	198 (173)	210 (171)	0.89
MMP-8/ TIMP-1	0.84 (1.78)	0.71 (1.79)	0.59	0.33 (0.76)	0.87 (1.80)	0.01	0.91 (1.59)	0.93 (1.80)	0.70	0.70 (1.33)	0.98 (1.62)	0.08
MMP-9/ TIMP-1	0.28 (0.65)	0.21 (0.69)	0.29	0.16 (0.74)	0.27 (0.60)	0.21	0.29 (0.54)	0.34 (0.66)	0.22	0.29 (0.74)	0.32 (0.53)	0.42

Significance of the differences calculated with Mann-Whitney´s U test.

## Discussion

We analyzed salivary levels of MMP-8 and -9, MPO, and TIMP-1 in MI and non-MI subjects with regard to their periodontal conditions. Salivary levels of MMP-8 were slightly higher in the non-MI group. This result differs to some extent, from earlier studies. Furuholm et al. [28] showed that patients referred for open heart surgery had significantly higher salivary concentrations of MMP-8 compared to matched controls after adjusting for number of teeth. No information about the reason for the open heart surgery or medical treatment was available. A more recent study showed a lower concentration of MMP-8 in saliva collected 3–4 days after the MI [27] compared to controls, which is in line with our findings. However, the same study also showed that the percentage of active MMP-8 was higher in the MI group. This differed from our study where a tendency of more active MMP-8 in the non-MI group was seen, although the same techniques and anti MMP-8 antibody were used as in Buduneli and co-authors study [27].

The reason for the discrepancy between the findings in our study and previous studies is not clear but possible explanations could be the time between the MI and saliva sampling, and the medication taken by the MI patients. The patients in this investigation were taking a number different medications, such as statins, angiotensin-converting enzyme inhibitors (ACEI), beta blockers, anti-inflammatory drugs and thrombocyte inhibitory agents (ASA) that could exert an effect on salivary levels of MMP-8 and -9, MPO and TIMP-1. According to previous studies ACEI and statins could potentially interfere with the expression of MPO, MMP-8 and -9. It has been described that ACE inhibitors can decrease MMP-9 levels in both acute and chronic phases of systemic conditions while mechanisms of statin use include inhibition of MMP-9 levels [29–31].

The present study did not only correlate salivary biomarkers to MI but also to the periodontal status. The clinical signs of periodontal inflammation, i.e. gingival inflammation (BOP) and PPD were more or less the same between the study groups. Three of the measured biomarkers, MMP-8, MMP-9 and MPO correlated with BOP, PPD and clinical periodontal diagnosis. TIMP-1 generally showed a negative correlation with the clinical signs of oral inflammation. These results are in agreement with resent studies [32, 33].

Yamamoto et al. showed that MMP-9 plays an important role in the onset and prognosis of MI [34]. In another study several inflammatory mediators and the relation to severity of CVD were studied in serum collected from patients with aortic sclerosis. Elevated general MMP expression was detected and MMP-9 and TIMP-1 had a positive correlation with each other [35]. In our current study MMP-9 correlated positively with MMP-8 and MPO but had a negative correlation with TIMP-1 levels. In previous animal studies, TIMP-1 was shown to inhibit both the activity of MMP-9 during infarct healing and in the protection against infarct plaque rupture after MI [36].

MPO in plasma is associated with an increased risk of CVD [37]. To our knowledge there are no studies of MPO in saliva in relation to MI but one cross-sectional study relating salivary MPO to ischemic stroke has been presented [33]. According to previous reports, MPO exerts distinct protective and surrogate roles during inflammation, such as inactivation of the pathogenic microbes and could oxidatively activate latent proMMP-8 and -9 and inactivate TIMPs [22, 38]. In fact non-proteolytic oxidative activation of proMMP-8 could be directly induced by MPO-derived hypochlorous acid, which is likely to represent the most direct mechanism for triggered neutrophils to endogenously activate MMP-8 [38]. Our results showed that MPO strongly correlated with MMP-8.

MMP-8 is currently regarded among the key biomarkers of inflammation [8, 39]. According to previous studies, salivary MMP-8 levels are higher in coronary artery disease patients and periodontitis patients [39, 40]. Furuholm and co-authors suggested that increased salivary levels of MMP-8 could reflect periodontal disease activity in patients with coronary artery disease compared to systemically healthy controls [28]. It has also been suggested that it may reflect cardiovascular disease, such as MI [28, 41].

It has been demonstrated that GCF levels of MPO and MMP-8 and associations between them were related to development and treatment responses in patients with chronic periodontitis. This indicates an interaction between the MPO oxidative pathway and MMP-8 activation and this cascade might be useful as a potential biomarker for assessing treatment outcomes [38].

The strong correlation between the measured salivary biomarkers and clinical conditions in the mouth illustrates the importance of considering oral inflammation when analyzing salivary biomarkers for systemic diseases. However, in the present study, MMP-8 and MPO were higher in the non-MI group also after compensation for periodontal inflammation and smoking, which might be explained, as previously discussed, by the extensive use of medication in the MI group at time of sample collection, 6–10 weeks after the MI.

In the current investigation, the analyzed biomarkers showed no relation with smoking. Smoking has been reported to increase as well as decrease levels of inflammatory mediators in oral fluids [16, 42]. The impact of smoking on salivary biomarkers of acute inflammation may be explained by a direct effect on inflammatory cells, both on their presence and activity and also the GCF volume per se. Smoking affect the expression and degranulation of MMPs and TIMP-1 [42, 43].

### Strengths and limitations of this study

Our finding regarding measured salivary enzymes (MMP-8, -9 and MPO), inhibitor (TIMP-1) and their molar ratios shows statistically significant associations between each other and to MI, as well as correlation to periodontal status. Salivary samples from patients were collected 6–10 weeks following the MI and the acute inflammatory response evoked by the MI had receded at this time. In addition, most patients were treated with CVD drugs at this time and this will likely have affected the analyzed biomarkers compared to if analysis had been performed at time of the MI.

## Conclusion

This study shows that salivary levels of the analyzed biomarkers are associated with periodontal status. However, these biomarkers could not differentiate between patients with or without a MI. These findings illustrate the importance to consider the influence of oral conditions when analyzing levels of inflammatory salivary biomarkers.

## Supporting Information

S1 Table All relevant data. (PDF)
