# Supplementary material for: Salivary Matrix Metalloproteinase-8 and -9 and Myeloperoxidase in Relation to Coronary Heart and Periodontal Diseases: A Subgroup Report from the PAROKRANK Study (Periodontitis and Its Relation to Coronary Artery Disease)
Source: PLoS One. 2015 Jul 1;10(7):e0126370. doi: 10.1371/journal.pone.0126370 (PMC4488442; doi:10.1371/journal.pone.0126370)
Supplement: S1 Table — (PDF) [file pone.0126370.s001.pdf]

Group	Sex	Age	Smoking	Snuffing	Hypertens	Diabetes	ACEI	ASA
0	1	61	1	0	0	0	1	1
0	1	64	1	0	0	0	1	1
0	1	62	1	0	0	0	0	1
0	1	50	3	0			0	1
0	1	61	3	0	1	0	1	1
0	1	63	1	1	1	0	1	1
0	1	50	1	1	1	0	0	1
0	1	62	3	0	1	0	0	1
0	1	65	3	0	1	0	1	1
0	1	40	1	0	0	0	1	1
0	1	49	1	0	0	0	0	0
0	1	52	1	1	0	0	0	1
0	1	45	1	0	0	0	1	1
0	1	41	1	1	1	0	0	1
0	1	67	1	1	1	0	1	1
0	1	65	1	0	0	0	1	1
0	2	53	1	0	1	0	1	1
0	1	49	1	0	0	0	1	1
0	1	62	3	0	0	0	1	1
0	2	63	1	0	0	0	0	1
0	1	58	1	0	0	0	1	1
0	2	67	1	0	0	0	0	1
0	1	65	3	0	0	9	0	1
0	1	64	3	0	0	0	1	1
0	1	64	1	0	1	1	0	1
0	1	65	1	0	1	0	1	1
0	1	67	1	0	1	0	1	1
0	1	65	2	0	1	0	1	1
0	1	58	3	0	1	0	0	1
0	1	47	2	0	1	0	0	1
0	1	47	1	1	0	0	0	1
0	1	55	3	0	0	0	1	1
0	1	51	2	2	0	0	1	1
0	1	70	3	0	0	0	0	1
0	1	58	3	0	0	0	1	1
0	1	57	3	0	1	0	1	1
0	2	66	3	0	0	0	0	1
0	2	65	1	0	1	0	1	1
0	1	61	1	0	1	0	0	0
0	1	66	1	1	0	0	0	0
0	1	44	1	0	0	0	0	0
0	1	63	1	0	0	0	0	0
0	2	55	1	0	0	0	0	1
0	2	64	4	0	1	1	0	1
0	2	72	1	0	0	0	0	0
0	1	68	1	0	0	0	0	0
0	1	59	3	0	1	1	0	1
0	1	72	3	0	0	0	0	0
0	1	54	3	0	0	0	0	0

0	1	56	2	0	0	0	0	0
0	1	63	3	2	0	0	0	0
0	1	56	1	0	0	0	0	0
0	1	66	2	0	0	1	0	0
0	1	59	3	0	1	9	1	1
0	1	62	1	2	0	0	0	0
0	1	55	3	0	0	0	0	0
0	1	62	3	0	0	0	0	0
0	1	60	3	0	0	0	0	0
0	1	55	3	0	0	0	0	0
0	1	74	3	0	0	0	0	0
0	2	64	1	0	0	0	0	0
0	1	63	1	1	0	0	0	0
0	2	53	2	0	0	0	0	0
0	1	50	1	1	0	0	0	0
0	1	67	1	0	1	0	1	1
0	1	46	1	1	0	1	0	1
0	2	72	1	0	0	0	0	0
0	1	71	3	0	0	0	0	0
0	1	73	3	0	0	0	0	0
0	1	57	3	0	0	0	0	0
0	1	64	1	0	0	0	0	0
0	1	60	1	1	1	0	0	1
0	1	53	3	0	1	0	1	1
0	1	44	2	2	0	0	0	0
0	1	65	1	0	1	0	0	0
0	1	74	1	0	1	0	0	1
0	1	69	1	0	0	0	1	1
0	1	28	2	0	0	0	1	1
0	1	70	1	0	1	0	1	1
0	1	70	1	0	0	0	1	1
0	1	65	2	2	1	1	0	1
0	2	68	3	0	1	1	0	1
0	1	52	3	0	0	1	0	1
0	1	60	1	0	1	0	0	1
0	1	63	2	2	0	0	0	1
0	1	69	3	0	0	0	0	1
0	1	70	3	0	1	0	1	1
0	1	62	2	0	1	0	1	1
0	1	67	4	2	1	0	0	1
0	1	71	2	0	0	0	1	1
0	1	48	2	0	0	0	1	1
0	1	60	2	1	1	0	1	1
0	2	56	3	0	0	1	1	1
0	1	64	1	0	0	0	0	1
0	2	68	1	0	0	0	0	1
0	1	64	1	1	0	0	0	1
0	2	68	3	0	0	0	1	1
0	1	57	1	0	0	0	1	1
0	1	62	1	0	0	0	0	1

0	1	65	2	0	0	0	1	1
0	2	56	3	0	1	0	1	1
0	1	68	2	0	0	0	1	1
0	1	59	1	0	1	1	1	1
0	1	63	1	0	1	0	1	1
0	1	56	1	0	1	0	1	1
0	1	70	1	0	0	0	1	1
0	2	65	3	0	1	0	1	0
0	1	69	3	0	0	0	0	0
0	1	63	3	0	1	0	1	0
0	2	67	1	0	0	0	0	0
0	1	46	1	1	1	1	0	0
0	1	67	1	1	0	0	0	0
0	1	65	1	0	1	0	0	0
0	1	65	3	0	0	0	0	0
0	1	59	1	0	1	0	1	0
0	1	62	1	0	0	0	0	0
0	1	60	1	1	1	0	0	1
0	1	56	1	0	0	0	0	0
0	1	57	2	2	0	0	0	0
0	1	48	3	0	0	0	0	0
0	1	60	2	0	0	0	0	0
0	2	68	1	0	0	0	0	0
0	1	50	1	0	1	0	0	0
0	1	63	2	0	1	0	1	0
0	1	63	3	0	1	0	0	0
0	1	68	1	0	1	0	1	0
0	1	73	3	0	0	0	0	0
0	1	56	3	0	1	0	1	0
0	2	61	1	0	0	0	0	0
0	1	51	1	0	1	0	0	0
0	1	65	1	0	9	0	0	0
0	1	64	1	0	0	0	0	0
0	1	67	1	0	0	0	0	0
0	1	50	1	1	1	1	1	0
0	1	61	3	0	0	0	0	0
0	1	67	1	1	0	0	0	0
0	1	65	1	0	1	0	1	1
0	1	72	3	0	1	0	0	0
0	1	52	4	1	0	0	1	1
0	1	69	1	0	0	0	0	1
0	1	64	1	0	1	0	1	1
0	1	57	1	0	0	0	1	1
0	1	70	1	0	0	0	1	1
0	2	70	3	0	0	0	0	1
0	1	38	1	1	1	0	1	1
0	1	60	1	0	1	0	1	1
0	1	66	2	0	0	0	0	1
0	1	57	1	0	0	0	0	1
0	1	71	1	0	0	0	1	1

0	1	56	3	0	1	0	1	1
0	1	68	3	0	9	0	1	1
0	2	70	1	0	0	0	1	1
0	1	62	1	0	0	0	1	1
0	1	59	1	0	0	0	1	1
0	1	61	1	0	1	0	1	1
0	2	63	3	0	1	0	1	1
0	1	67	1	0	1	0	0	1
0	1	47	1	0	0	0	1	1
0	2	73	3	0	1	1	0	1
0	2	53	2	0	0	1	1	1
0	2	67	3	0	0	0	1	1
0	1	55	3	0	0	1	0	1
0	1	65	1	0	0	0	1	1
0	1	63	3	0	1	1	0	1
0	1	67	1	1	1	1	0	1
0	1	66	3	0	0	0	1	1
0	1	65	3	0	1	1	1	1
0	1	68	1	2	0	0	1	1
0	1	73	2	0	1	0	0	1
0	1	62	1	0	0	0	1	1
0	1	65	3	0	1	0	1	1
0	1	68	1	0	1	0	1	1
0	1	73	3	9	0	0	1	1
0	1	61	1	1	0	0	1	1
0	1	62	2	0	0	0	1	1
0	1	68	1	0	1	0	1	1
0	2	73	1	0	0	0	0	0
0	1	51	1	0	1	0	0	0
0	1	53	1	1	1	0	0	0
0	1	67	3	0	0	0	0	0
0	1	58	1	0	0	0	0	0
0	1	71	1	0	0	0	0	0
0	2	70	1	0	1	0	0	1
0	1	60	1	1	1	0	1	0
0	1	63	1	0	0	0	0	0
0	1	65	1	0	1	0	0	0
0	1	55	3	0	1	0	0	1
0	1	60	1	1	1	0	0	0
0	2	73	1	0	0	0	0	0
0	1	44	3	0	1	0	1	0
0	1	68	4	0	9	0	0	0
0	1	58	1	0	0	0	0	0
0	1	63	1	0	1	0	0	0
0	1	61	1	1	1	0	0	1
0	2	57	1	0	0	0	0	0
0	2	65	1	0	1	0	0	1
0	1	56	1	0	1	0	0	1
0	1	74	1	0	0	0	0	0
0	1	74	3	0	1	0	0	0

0	1	74	1	0	0	0	0	0
1	1	61	1	0	0	0	0	0
1	1	64	1	0	0	0	0	0
1	1	61	1	0	1	0	0	0
1	1	50	2	0	1	0	0	0
1	1	61	3	0	0	0	0	0
1	1	63	3	0	1	0	1	0
1	1	50	4	1	0	0	0	0
1	1	63	1	0	0	0	0	0
1	1	65	3	0	1	0	0	0
1	1	41	3	0	0	0	0	0
1	1	50	2	2	0	0	0	0
1	1	53	3	0	0	0	0	0
1	1	45	2	2	0	0	0	0
1	1	41	3	0	0	0	0	0
1	1	66	2	2	1	0	0	1
1	1	65	1	1	1	0	1	1
1	2	54	2	0	0	0	1	1
1	1	49	3	0	1	0	1	1
1	1	62	3	0	0	0	1	1
1	2	63	1	0	0	0	1	1
1	1	59	1	1	1	0	1	1
1	2	67	2	0	1	0	0	1
1	1	66	1	0	0	0	0	1
1	1	65	1	0	0	0	1	1
1	1	65	1	0	0	0	0	1
1	1	66	1	0	1	1	1	1
1	1	68	3	0	0	0	1	1
1	1	66	3	0	0	0	1	1
1	1	59	2	0	0	0	0	1
1	1	47	1	1	0	0	0	1
1	1	47	3	0	1	0	1	0
1	1	55	3	0	0	0	0	0
1	1	51	1	0	0	0	0	0
1	1	70	1	0	0	0	0	0
1	1	58	1	0	0	0	0	0
1	1	57	1	0	0	0	0	0
1	2	66	1	0	1	0	0	0
1	2	65	3	0	1	0	0	1
1	1	62	3	0	0	0	0	0
1	1	67	3	0	1	1	1	0
1	1	45	2	0	0	0	0	0
1	1	64	3	0	0	0	0	0
1	2	55	4	0	1	0	0	1
1	2	64	3	0	0	0	0	0
1	2	72	3	0	0	0	0	0
1	1	68	3	0	0	0	0	0
1	1	59	1	1	0	0	0	0
1	1	72	1	0	0	0	1	0
1	1	54	3	0	1	0	0	1

1	1	56	3	0	0	0	1	1
1	1	63	1	0	1	0	1	1
1	1	57	4	0	0	0	1	1
1	1	66	1	1	0	0	1	1
1	1	59	1	1	1	0	1	1
1	1	62	1	0	0	0	1	1
1	1	54	2	2	0	0	1	1
1	1	63	1	0	1	1	0	1
1	1	61	3	0	0	0	1	1
1	1	56	1	0	1	0	1	1
1	1	75	1	0	1	0	1	1
1	2	65	1	0	1	0	0	1
1	1	63	1	1	1	0	1	1
1	2	53	3	0	1	0	1	1
1	1	50	3	0	0	0	1	1
1	1	67	1	0	1	0	1	1
1	1	46	1	0	1	0	1	1
1	2	72	3	0	1	0	1	1
1	1	71	1	0	0	0	0	0
1	1	74	2	0	1	0	0	0
1	1	57	1	1	0	0	0	0
1	1	64	3	0	0	0	0	0
1	1	60	1	1	0	0	0	1
1	1	53	2	2	0	0	0	0
1	1	44	3	0	1	0	0	0
1	1	65	3	0	0	0	0	0
1	1	74	1	0	0	0	0	0
1	1	69	3	0	0	0	0	0
1	1	28	4	9	0	0	0	0
1	1	70	1	0	0	0	0	0
1	1	70	1	1	0	0	0	0
1	1	65	2	2	1	0	0	1
1	2	68	3	0	0	0	0	0
1	1	52	3	0	1	0	1	0
1	1	60	2	2	0	0	0	0
1	1	63	1	0	0	0	0	0
1	1	69	1	1	0	0	0	0
1	1	70	1	0	0	0	0	0
1	1	62	3	0	1	0	1	1
1	1	67	1	0	0	0	1	1
1	1	71	3	0	1	1	1	1
1	1	49	4	0	0	0	0	1
1	1	60	1	1	0	0	1	1
1	2	57	1	0	0	0	1	1
1	1	65	3	0	0	0	1	1
1	2	68	1	0	1	0	1	1
1	1	64	1	1	0	0	1	1
1	2	68	1	0	0	0	1	1
1	1	62	1	0	0	0	1	1
1	1	62	3	0	1	0	0	1

1	1	65	4	0	0	0	1	1
1	2	56	2	0	0	0	0	1
1	1	67	2	2	1	0	0	1
1	1	59	3	0	0	0	0	1
1	1	63	2	2	1	0	1	1
1	1	56	3	0	0	0	0	1
1	1	70	3	0	1	0	1	1
1	2	65	3	0	0	0	0	1
1	1	68	1	0	0	0	1	1
1	1	63	1	1	0	0	1	1
1	2	67	2	0	0	0	1	1
1	1	45	3	0	1	0	0	1
1	1	67	1	2	0	0	0	1
1	1	65	1	0	0	0	0	0
1	1	65	2	0	0	0	0	0
1	1	59	1	2	0	0	0	0
1	1	62	1	2	0	1	0	0
1	1	61	1	0	1	0	1	0
1	1	56	3	0	0	0	0	0
1	1	58	1	0	0	0	0	0
1	1	49	3	0	0	0	0	0
1	1	60	1	0	1	0	1	0
1	2	68	1	0	1	0	1	0
1	1	50	1	0	0	0	0	0
1	1	63	1	1	1	0	0	0
1	1	63	1	1	1	0	1	0
1	1	68	3	0	1	0	1	1
1	1	73	1	0	0	0	0	0
1	1	56	3	0	0	0	0	1
1	2	62	4	0	1	0	0	1
1	1	52	1	1	0	0	0	0
1	1	66	1	0	0	0	0	0
1	1	65	3	0	0	0	0	0
1	1	67	3	0	0	0	0	0
1	1	50	3	0	0	0	0	0
1	1	61	1	1	0	0	0	0
1	1	67	3	0	0	0	0	0
1	1	65	3	0	0	0	0	0
1	1	72	1	0	1	0	0	0
1	1	52	4	1	0	0	1	1
1	1	69	4	2	1	0	1	1
1	1	64	1	1	1	0		
1	1	58	3	0	0	0	1	1
1	2	70	1	0	0	0	1	1
1	2	71	1	0	0	0	1	1
1	1	39	2	2	0	0	0	1
1	1	60	2	1	0	0	0	0
1	1	66	2	0	0	0	0	0
1	1	57	1	0	1	1	0	1
1	1	71	1	0	1	0	0	0

1	1	56	3	0	1	0	0	0
1	1	68	1	0	1	0	0	0
1	2	70	3	0	1	0	0	1
1	1	62	2	2	0	0	1	1
1	1	59	3	0	0	0	1	1
1	1	62	1	0	0	0	1	1
1	2	63	1	0	1	0	1	9
1	1	67	3	0	1	0	1	1
1	1	48	2	2	0	0	1	1
1	2	73	3	0	1	1	1	1
1	2	54	1	0	1	0	0	1
1	2	68	2	0	0	0	1	1
1	1	56	3	0	0	0	1	1
1	1	66	1	0	0	0	0	1
1	1	68	2	2	0	0	0	1
1	1	68	2	0	0	0	1	1
1	1	67	3	0	0	0	1	1
1	1	66	1	0	1	1	1	1
1	1	68	2	2			0	1
1	1	73	1	0	0	0	1	1
1	1	62	2	2	1	0	0	1
1	1	65	2	1	0	0	1	1
1	1	68	1	0	1	0	1	1
1	1	73	1	0	0	0	0	0
1	1	61	1	1	0	0	0	0
1	1	63	3	0	0	0	0	0
1	1	69	3	0	0	0	0	0
1	2	74	1	0	1	0	0	0
1	1	51	3	0	1	0	0	0
1	1	55	3	0	0	0	0	1
1	1	67	3	0	0	0	0	0
1	1	58	2	2	1	0	0	0
1	1	71	3	0	1	0	0	0
1	2	70	3	0	0	0	0	0
1	1	60	2	0	0	0	0	0
1	1	63	2	0	0	0	0	0
1	1	65	1	0	0	0	0	0
1	1	55	2	2	0	0	0	0
1	1	60	2	0	0	0	0	0
1	2	73	2	2	0	0	0	0
1	1	44	3	0	1	0	0	0
1	1	68	3	0	0	0	0	0
1	1	58	3	0	0	0	0	0
1	1	63	1	0	1	0	0	1
1	1	61	2	2	1	1	1	1
1	2	57	2	2	0	0	1	1
1	2	65	1	0	1	0	1	1
1	1	56	1	0	0	0	0	0
1	1	74	1	0	0	0	0	0
1	1	74	1	1	1	1	0	0

1	1	74	3	0	0	0	0	0

Beta block	Statins	Plaque	BOP	PPD 4–5 r	PPD ≥ 6 n	Total PPD	MMP-8	MMP-9
1	1	54	45	19	3	108	530,55	330,53
1	1	48	47	14	0	57	506,5	330
1	1	49	22	12	1	42	435,91	207,38
1	1	103	71	2	0	8	99,06	26,37
1	1	123	76	20	0	53	740,8	380,94
1	1	81	68	20	0	84	662,38	114,58
1	1	76	58	18	2	89	418,05	251,16
1	1	46	6	4	0	8	106,34	221,7
1	1	113	45	7	0	20	727,44	68,18
1	1	108	92	19	0	79	433,93	474,88
1	1	67	75	40	11	243	396,2	629,54
1	1	85	10	0	0	0	387,65	44,13
1	1	65	28	24	10	162	708,5	520,13
1	1	104	58	40	5	202	88,17	139,79
1	1	112	0	0	0	0	193,65	284,13
1	1	96	50	6	1	30	438,69	190,77
1	1	24	23	2	0	8	140,92	9,36
1	1	117	25	0	0	1	294,27	128,9
1	1	83	80	8	0	20	243,99	61,95
1	1	25	20	5	1	26	303,51	314,49
1	0	68	68	10	1	47	690,69	320,22
1	1	53	80	6	0	25	169,73	57,87
1	1	42	8	3	0	4	551,96	272,68
1	1	33	12	4	0	13	40,99	101,98
1	1	75	87	23	0	83	830	296,74
1	1	65	62	10	0	42	365,73	131,77
1	1	54	15	41	2	196	871,15	280,7
1	1	48	44	5	43	440	724,8	612,93
1	1	64	7	5	0	16	55,7	3,46
1	1	104	104	56	26	349	623,79	111,72
1	1	88	68	0	0	0	902,15	317,36
1	1	16	2	0	0	0	469,24	204,51
1	1	78	8	16	0	59	416,53	186,76
1	1	14	1	0	0	0	208,84	110
1	1	86	59	59	0	225	739,45	130,14
1	1	64	57	42	0	170	1049,65	369,42
1	1	28	2	0	0	1	202,85	67,61
1	1	0	1	0	0		61,28	110,57
1	0	87	75	7	0	32	308,52	257,21
0	0	75	24	17	1	95	499,63	150,1
0	0	84	13	6	1	35	193,83	77,92
0	0	50	55	27	6	158	186,23	222,27
0	0	20	20	9	6	65	284,27	239,45
1	1	16	16	1	0	4	159,98	5,17
0	0	84	38	2	0	10	27,47	58,55
0	1	76	3	0	0	0	19	0
0	1	119	21	0	0	0	83,64	233,05
0	0	75	8	0	0	4	76,33	118,25
1	0	18	9	3	0	4	342,54	213,15

0	0	76	65	47	7	244	187,42	8,03
0	0	88	76	2	0	9	152,87	584,36
0	0	68	34	12	1	50	206,76	59,31
0	1	112	51	33	3	149	324,64	51,66
1	1	85	80	18	0	65	250,05	686,92
0	0	95	61	18	1	74	1186,49	90,69
0	0	77	84	0	0	0	136,07	305,76
0	0	66	82	16	0	60	610,32	481,03
0	0	57	76	5	2	34	274,11	86,1
0	0	49	34	2	0	8	1531,62	1338,25
0	1	54	7	7	0	28	299,94	226,16
0	0	0	0	0	0	0	4,22	0
0	0	15	2	0	0	0	18,47	26,4
0	0	21	8	46	1	189	30,33	0
0	0	44	0	0	0	0	208,46	82,28
0	1	78	31	20	12	0	102,26	314,18
1	1	80	76	3	0	8	462,04	612,68
0	0	41	5	17	1	0	225,83	295,81
0	0	94	4	0	0	0	444,72	98,35
0	0	88	1	1	0	0	155,11	8,03
0	0	56	27	1	0	4	649,26	87,63
0	0	101	51	19	2	84	1148,29	404,5
1	1	59	66	15	2	63	764,76	677,73
0	1	60	24	6	2	37	309,65	163,4
0	0	45	70	3	0	12	393,69	317,24
0	0	35	35	22	5	128	526,7	350,15
0	0	23	15	10	1	48	27,46	55,49
1	1	42	72	18	3	103	335,14	803,25
1	1	76	67	14	1	52	575,81	344,8
1	1	88	52	16	3	88	512,99	907,34
1	1	21	12	5	0	23	494,21	146,57
1	1	32	48	17	9	103	288,96	264,43
1	1	80	53	14	2	65	983,86	216,22
1	1	39	29	0	0	0	72,54	84,57
1	1	9	21	3	0	4	16,01	1,15
1	1	88	62	20	11	130	29,32	661,16
1	0	68	57	6	1	35	47,74	131,96
1	1	88	52	12	1	41	562,35	176,26
1	1	77	74	39	13	252	320,24	104,17
1	1	42	20	7	1	34	153,99	325,49
1	1	3	44	28	11	192	155,39	399,82
0	0	102	51	7	0	13	174,42	35,99
1	1	77	23	13	0	56	147,27	188,84
1	1	73	46	6	0	25	18,02	138,54
1	1	100	99	17	3	92	931,27	145,07
1	1	61	43	6	0	25	80,8	264,61
1	1	73	81	21	1	98	1110,25	261,34
1	1	18	6	5	0	21	24,57	0
1	1	80	28	7	6	38	196,67	138,54
1	1	119	74	16	0	50	14,8	24,88

1	1	60	53	11	2	61	1081,56	605,59
1	1	61	43	7	2	43	292,13	106,53
1	1	65	22	23	0	85	148,42	137,89
1	1	72	67	3	3	32	167,58	144,42
0	1	84	22	11	4	68	773,86	34,03
1	0	72	41	33	5	182	115,1	164,02
1	1	74	64	6	4	42	725,46	287,47
1	1	58	90	21	1	81	239,59	582,27
0	0	19	6	10	1	40	1089,15	783,26
1	0	27	15	13	2	71	179,41	46,44
0	0	4	11	22	5	128	1079,71	828,98
1	1	60	34	9	0	37	114,68	5,94
0	0	50	19	20	1	88	528,62	545,49
0	1	57	47	33	7	186	1242,76	1330
0	0	60	2	4	0	17	84,33	3,33
0	0	59	12	9	1	43	630,64	544,18
0	0	32	19	19	1	93	87,49	233,91
1	0	67	18	11	1	38	746,21	585,34
0	0	14	9	0	1	6	105,28	334,5
0	0	43	28	29	0	117	32,91	235,87
0	0	55	35	27	4	125	3,07	0
0	0	74	32	39	7	192	476,12	107,19
0	0	57	10	20	2	99	43,37	293,35
1	0	34	21	9	3	66	615,9	383,49
0	0	67	18	37	34	357	772,45	188,84
1	0	21	22	0	0	44	379,97	121,56
0	0	56	37	9	2	48	948,06	103,27
0	0	11	35	6	3	5	1192,6	1721,27
1	0	10	6	1	0	0	1086,27	410,93
0	0	2	1	0	0	0	230,82	148,99
0	1	9	19	0	0	0	763,37	231,33
0	0	24	18	0	0	82	1190,98	469,84
0	0	54	38	12	2	0	1108,78	475,33
0	0	45	38	0	0	0	291,07	168,78
0	1	24	21	0	0	0	625,49	119,98
0	1	45	48	0	0	0	1211,54	85,61
0	0	29	17	0	0	0	818,75	139,91
0	1	7	14	0	0	0	720,2	274,63
0	0	6	2	0	0	0	240,9	51,25
1	1	8	32	0	0	0	1227,4	388,73
0	1	52	48	0	0	0	1126,41	32,69
1	1	30	26	0	0	0	1027,35	188,03
1	1	47	39	0	0	0	1336,11	119,29
1	1	3	2	0	0	0	235,48	317,93
1	0	19	3	1	0	130	276,62	401,1
1	0	51	74	41	0	179	642,21	559,88
1	1	25	30	38	3	120	251,65	129,6
1	0	55	51	17	8	123	844,82	390,1
1	1	65	28	36	1	12	635,24	525,51
1	1	8	12	3	0	125	90,82	178,4

1	1	63	20	43	0	85	777,38	367,42
1	1	29	21	25	0	49	542,75	852
1	1	11	2	12	0	102	298,85	104,17
1	1	26	21	22	1	294	252,8	644,42
1	1	33	23	33	0	41	613,66	199,02
1	1	15	9	11	0	29	547,21	150,22
1	1	17	21	7	0	32	182,21	294,56
1	1	24	14	8	0	343	142,14	49,18
1	1	78	70	72	3	79	969,44	270,51
1	1	34	35	19	0	191	801,07	465,71
1	1	37	47	38	9	33	35,55	144,04
1	1	30	16	8	0	106	780,93	520,7
1	1	63	43	29	0	60	237,85	27,19
1	1	30	26	19	0	126	161,08	168,09
1	0	41	26	34	1	188	14,46	158,47
1	1	63	96	32	13	43	1025,94	897,36
1	1	23	15	9	1	95	228,1	267,76
1	1	19	21	21	1	0	430,39	236,14
0	1	0	0	0	0	195	53,42	294,56
1	1	42	9	46	0	85	866,15	136,48
1	1	42	44	13	0	10	662,38	63,98
1	1	32	9	2	0	42	28,07	172,01
1	1	33	0	10	0	64	6,45	0,26
1	1	35	24	16	0	61	19,78	82,19
1	1	0	7	19	0	120	10,54	86,44
1	1	11	5	22	4	0	954,32	630,82
1	1	0	0	0	0	0	64,57	0
0	0	50	22	20	2	87	1327,37	852,94
0	1	46	13	23	1	97	56,51	40,32
0	0	70	31	29	0	48	1218,53	247,27
0	0	22	26	22	0	101	283,75	286,11
0	0	47	21	21	2	0	675,77	297,64
0	0	0	0	0	0	96	266,73	186,58
0	1	93	22	23	1	98	19,52	0
0	0	93	29	20	2	105	1223,56	335,87
0	0	112	27	26	0	91	49,15	0
1	0	32	31	22	0	70	128,55	91,9
1	1	30	17	20	0	212	28,81	36,07
1	1	77	30	31	12	64	1375,88	736,42
0	0	16	16	16	0	60	69,51	67,02
1	1	90	11	22	0	68	257,44	351,65
0	0	83	18	17	0	169	45,08	253,33
0	0	76	41	42	0	42	729,43	267,9
0	0	35	5	10	0	118	39,82	37,28
1	1	46	32	33	0	84	492,86	255,76
0	0	67	23	22	0	57	725,46	470,6
0	1	40	22	9	3	77	116,57	80,37
0	1	10	4	16	0	40	63,18	23,32
0	0	11	9	10	0	26	79,34	82,19
0	0	12	14	6	0	57	957,87	63,98

0	0	7	11	16	0	54	219,16	188,4
0	0	19	4	0	0	0	617,02	173,58
0	0	92	91	10	0	36	556,24	282,7
0	0	52	24	14	1	63	823,23	87,66
0	1	30	22	9	0	37	597,14	46,42
0	1	42	26	29	2	139	342,54	177,02
0	0	24	19	12	0	40	932,91	93,39
0	1	116	60	11	1	24	1135,11	524,95
0	0	74	30	11	1	53	416,15	128,9
1	0	60	14	16	0	58	213,35	347,72
0	0	107	99	3	0	13	518,62	283,56
0	0	44	70	2	0	12	403,84	288,14
0	0	118	14	9	6	49	713,03	446,81
0	0	79	13	1	0	7	863,33	193,06
0	0	33	2	0	0	0	477,85	57,87
1	1	104	19	4	2	27	107,9	252,63
1	1	33	2	4	0	16	643,38	308,19
1	0	26	3	0	0	12	92,44	0
1	1	46	22	1	0	0	161,59	206,23
1	1	33	10	0	0	37	1047,66	217,47
0	1	77	18	23	4	124	603,15	195,92
1	1	35	6	6	0	12	150,46	68,18
1	1	36	2	19	1	79	52,88	0
1	1	29	7	0	0	22	815,78	421,61
1	1	73	48	9	0	39	420,72	245,76
1	1	46	8	0	0	3	413,51	226,28
1	1	94	76	50	11	196	953,23	639,28
1	1	105	15	5	0	17	391,55	154,11
1	1	42	0	0	0	0	351,7	85,94
0	0	60	121	32	14	229	974,16	6,89
1	1	113	0	0	0	0	557,26	468,01
1	1	67	0	0	0	0	123,56	49,85
0	0	45	2	0	0	0	27,27	33,82
0	0	39	0	0	0	0	88,17	52,72
0	0	2	0	0	0	0	229,14	405,57
0	0	108	85	54	54	590	1185	228
0	0	14	0	0	0	0	67,64	85,6
0	0	34	0	0	0	0	66,9	52,15
0	0	49	55	0	0		82,66	249,76
0	0	94	63	8	0	24	384,49	88,23
0	0	78	55	27	1	84	795,99	202,22
0	0	100	94	4	0	17	1433,29	184,46
0	0	50	11	2	0	4	179,16	189,05
1	1	25	6	0	0	0	102,17	91,67
0	0	7	21	2	0	0	815,04	323,08
0	0	42	29	8	0	32	583,72	409,85
0	0	47	8	0	0	0	746,21	425,16
0	0	21	9	0	0	0	219,95	405,26
1	1	7	3	2	1	16	133,25	370,05
1	1	68	11	0	0	0	1233,03	879,79

1	1	54	2	2	0	8	91,98	128,2
1	1	17	9	12	0	47	286,87	350,15
1	1	36	8	0	0	0	678,24	309,59
1	1	62	1	2	0	8	573,19	86,87
1	1	50	17	0	0	0	416,15	135,85
1	1	60	6	2	0	8	114,99	112,12
0	1	46	15	2	0	0	87,33	164,94
1	1	95	65	8	0	22	1475,49	637,17
1	1	47	68	8	0	35	788,78	167,23
1	1	68	52	5	0	20	474,82	288,16
1	1	110	53	3	0	16	318,79	304,23
1	1	36	0	0	0	0	87,45	117,48
1	1	74	0	0	0	0	197,12	288,16
1	1	82	2	0	0	0	307,12	301,17
1	1	84	82	0	0		164,26	193,25
1	1	82	2	0	0		375,84	251,42
1	1	64	38	39	6		850	483,33
1	1	76	46	37	14		428,05	214,68
0	0	104	52	58	3		532,48	99,11
0	0	100	34	92	7		848,52	40,95
1	0	76	73	7	5	63	1047,09	374,11
0	0	73	77	16	8	108	1188,51	623,39
0	0	94	31	23	3	95	449,19	431,28
0	0	43	23	24	0	99	934,14	200,91
1	0	74	77	9	0	24	917,37	368,52
0	0	43	38	0	0	0	849,44	393,02
0	0	36	21	8	0	33	265,77	604,26
0	0	103	58	7	3	51	1353,97	426,69
0	0	49	43	2	0	0	1112,66	301,94
0	0	55	54	21	0	89	1329,24	377,71
0	0	87	68	35	4	173	1362,17	256,01
1	0	74	54	20	2	100	351,06	62,38
0	0	18	6	0	0	0	60,81	153,8
0	0	16	46	4	0	16	65,67	267,49
0	0	104	79	12	2	64	215,6	60,08
0	1	83	55	11	1	55	462,88	388,06
0	0	105	83	11	1	55	561,33	441,47
1	1	0	0	10	0	40	60,28	116,18
0	1	68	69	32	9	160	645,73	337,87
1	1	50	29	5	2	32	210,94	228,58
0	1	80	56	24	3	103	262,88	416,15
1	1	70	49	20	0	84	403,84	162,06
0	1	86	39	5	0	22	212,48	292,05
1	1	76	21	14	2	60	157,03	359,98
1	1	71	40	3	3	32	117,21	182,96
1	1	65	6	5	0	20	31,53	47,74
1	1	72	34	11	2	55	475,68	452,73
1	1	56	59	10	4	70	603,15	0
1	1	73	34	11	4	75	600,41	299,23
1	1	77	31	2	0	9	846,29	353,45

1	1	24	38	13	0	59	50,65	94,78
1	1	24	16	4	0	18	52,49	16,04
1	1	77	29	15	2	79	840,76	337,12
1	1	69	30	1	0	0	661,78	596,44
1	1	75	61	9	1	52	1062,2	33,64
0	0	12	20	9	4	68	192,95	0,26
1	1	90	49	9	1	43	45,54	222,15
1	1	54	31	11	5	84	362,08	713,37
1	1	27	14	12	0	53	845,76	331,89
1	1	19	15	3	1	18	241,12	253,51
1	1	32	10	4	1	23	1065,36	1220,91
0	1	21	34	18	2	75	426,11	163,36
0	1	22	10	13	0	58	785,92	287,47
0	0	82	20	20	0	84	1312,51	313,6
0	0	37	12	5	1	29	627,2	250,89
0	0	50	23	12	3	69	342,54	326,01
0	0	18	3	1	0	4	41,11	54,93
0	0	58	6	12	3	73	740,12	299,88
0	0	68	31	15	0	60	451,65	245,67
0	0	16	16	5	0	20	128,37	250,89
0	0	49	28	7	0	28	18,36	0
0	1	18	20	3	0	95	312,47	276,37
0	0	29	5	12	6	9	1090,89	551,37
0	0	28	7	2	0	97	270,15	224,76
0	0	40	16	15	5	0	768,94	8,55
1	0	26	21	0	0	9	1071,64	712,06
1	0	12	24	0	1	0	1076,15	1491,99
0	0	9	4	0	0	0	195,78	104,57
0	0	12	6	0	0	0	21,49	103,92
0	0	16	8	0	0	3	33,14	0
0	0	34	57	0	0	0	545,22	52,62
0	0	21	7	0	0	0	817,26	491,14
0	0	15	18	0	0	0	780,22	258,14
0	0	30	28	2	0	0	1112,85	245,08
0	0	71	86	0	0	20	1104,19	170,16
0	0	61	43	4	0	0	1025,29	64,31
0	0	16	15	2	2	0	903,06	457,46
0	0	8	8	0	0	0	1078,84	479,46
0	0	12	0	0	0	10	275,61	267,07
1	1	57	57	1	0	10	1163,13	185,96
1	1	55	53	2	0	0	568,52	138,54
		19	6	0	0	0	981,51	287,69
1	1	56	56	0	0	10	946,39	215,52
1	1	26	21	2	0	4	378,59	240,26
1	1	41	20	21	0	36	45,64	285,63
0	1	20	18	12	0	124	409,76	322,06
0	0	34	43	35	0	117	702,09	152,28
0	0	23	11	11	10	202	384,49	497,33
1	1	46	40	51	0	78	744,17	513,14
0	0	33	45	23	0	53	833,72	258,82

0	1	7	7	15	0	146	12,88	15,5
0	0	39	31	25	6	37	496,46	169,47
0	0	22	14	11	0	178	488,4	497,33
1	1	30	22	41	0		337,9	244,39
1	1	20	20	20	0	64	152,59	111,73
1	1	31	31	16	0	59	299,4	117,92
0	1	45	57	17	0	238	669,65	0
1	1	47	55	37	14	209	1132,07	0
1	1	79	90	51	0	50	1793,37	385,98
1	1	22	33	25	0	48	87,45	455,4
1	1	1	17	12	0	93	105,96	379,11
1	1	54	22	23	0	57	666,62	95,92
0	1	53	27	12	1	40	345,99	241,2
1	1	47	8	10	0	90	143,7	344,05
1	1	56	24	27	0	34	278,13	301,44
1	1	12	13	5	2	22	394,41	205,9
0	1	44	12	6	1	171	1163,18	40,25
1	1	60	37	38	1	92	763,37	463,65
1	1	15	19	13	0	98	103,48	351,61
1	1	12	13	21	2	56	43,77	469,15
1	1	20	20	0	0	66	200,65	237,56
1	1	17	23	15	1	218	44,71	131,96
1	1	17	27	52	0	114	412,38	766,76
0	0	44	4	28	0	41	103,06	477,88
0	0	39	2	7	2	124	12,71	89,47
0	0	52	20	35	2	267	1137,65	130,14
0	0	61	26	60	3	93	10,58	179,29
0	0	30	15	21	0	64	687,55	380,78
0	0	51	18	18	0	130	255,57	36,07
1	1	19	6	32	0	117	800,34	220,56
0	0	98	18	28	0	57	1054,18	684,83
0	1	61	18	16	0	28	350,42	581,66
0	0	74	21	9	0	0	546,22	289,75
0	0	93	40	13	2	271	866,02	133,17
0	0	52	51	43	12	259	1228,49	31,21
1	0	74	43	62	0	158	722,83	11,79
0	0	31	45	43	0	84	783,06	409,91
1	0	112	28	26	0	214	223,99	124,67
0	0	32	43	34	9	219	746,89	16,65
0	0	31	48	41	6	56	24,73	0
1	0	23	19	15	0	24	873,35	635,68
0	1	42	9	10	0	80	114,32	8,15
0	0	26	17	20	0	56	26,65	0,87
1	1	18	17	14	0	53	1079,57	25,75
0	1	51	23	19	0	143	41,74	64,59
0	1	38	32	28	3	50	1141,94	209,64
1	1	26	18	12	0	64	992,52	56,1
0	0	18	21	18	0	64	1190,41	953,69
0	0	58	10	17	0	68	786,63	173,23
1	1	10	10	17	0	42	1235,44	26,36

0	0	0	10	10	0	26	468,81	659,95

MPO	TIMP-1	MMP-8/ T	MMP-9/ T	Pro MMP-	Active MMP-8
848,5	270,4	0,85	0,37	7,52	23,42
4419,8	172,53	1,26	0,58	8,95	37,04
1610,4	107,04	1,75	0,59	9,43	35,17
1594,5	173,78	0,25	0,05	6,99	25,34
3658	69,52	4,59	1,67	8,92	33,61
2777	128,46	2,22	0,27	8,7	33,92
705,7	214,56	0,84	0,36	8,09	25,52
0	157,98	0,29	0,43	8,08	18,82
4070,6	59,76	5,24	0,35	9,89	39,99
3904	85,1	2,2	1,7	9,6	33,4
1558,8	83,23	2,05	2,3	8,96	20,32
221,6	308,47	0,54	0,04	8,05	12,23
3554,8	38,67	7,89	4,09	11,41	8,95
237,5	577,41	0,07	0,07	6,08	0
427,9	175,5	0,48	0,49	3,94	0
1094,6	207,22	0,91	0,28	7,21	6,85
0	226,19	0,27	0,01	7,42	0
213,6	223,76	0,57	0,18	7,92	0,53
1376,3	168,07	0,63	0,11	8,16	3,14
257,3	179,14	0,73	0,53	9,84	8,93
1078,7	110,98	2,68	0,88	9,5	5,57
51	209,71	0,35	0,08	9,65	19,71
1626,3	90,53	2,63	0,92	9,64	23,55
0	143,41	0,12	0,22	7,53	10,35
1372,3	115,92	3,08	0,78	9,56	21,02
420	57,97	2,72	0,69	7,52	12,8
1562,8	81,59	4,6	1,05	10,58	23,99
3784,9	28,77	10,85	6,48	7,32	39,87
447,8	186,68	0,13	0,01	3,98	31,89
4542,8	35,43	7,58	0,96	8,59	42,82
2269,1	190,02	2,05	0,51	6,01	42,83
1507,2	231,51	0,87	0,27	3,97	14,81
987,4	118,02	1,52	0,48	8,32	3,33
344,6	157,11	0,57	0,21	4,25	1,58
1789	224,94	1,42	0,18	6,98	21,35
4836,5	106,45	4,25	1,06	11,38	19,17
1412	169,19	0,52	0,12	7,39	13,89
253,3	169,32	0,16	0,2	7,61	2,52
590,6	146,09	0,91	0,54	10,85	11,8
396,4	159,22	1,35	0,29	7,75	14,51
407	147,33	0,57	0,16	6,84	1,6
670,8	104,67	0,77	0,65	11,72	7,91
494	141,92	0,86	0,51	8,7	10
662,5	235,97	0,29	0,01	10,88	7,49
304,7	113,71	0,1	0,16	5,11	5,43
360,6	235,99	0,03	0	4,48	4,13
269,8	198,1	0,18	0,36	2,08	24,33
647	270,7	0,12	0,13	6,43	9,94
1032,2	209,99	0,7	0,31	10,42	23,87

647	200,03	0,4	0,01	5,94	5,76
1496,5	141,51	0,47	1,26	10,21	20,24
802,7	144,04	0,62	0,13	0,62	0
1003,2	235,85	0,59	0,07	6,7	0
1966,1	43,72	2,46	4,78	14,64	5,8
2731,1	55,64	9,19	0,5	11,93	17,13
362,1	180,73	0,32	0,51	12,54	13,74
457,1	73,85	3,56	1,98	12,2	12,57
684	134,63	0,88	0,19	10,54	11,37
1995,1	95,69	6,89	4,26	17	47,53
834,3	98,84	1,31	0,7	13,45	29,15
0	172,64	0,01	0	2,85	37,35
0	170,83	0,05	0,05	8,93	8,47
348,9	380,96	0,03	0	3,96	8,68
916,1	133,1	0,67	0,19	8,71	23,12
641,8	82,99	0,53	1,15	10,16	22,64
1092,9	55,45	3,59	3,36	13,76	32,08
549,4	271,93	0,36	0,33	11,03	30,95
1330,3	267,42	0,72	0,11	12,55	40,24
40,3	209,4	0,32	0,01	8,01	15,8
2148,1	277,29	1,01	0,1	14,19	16,09
3820,7	42,06	11,76	2,93	19,74	34,63
2224,6	40,95	8,05	5,04	19,6	19,99
855,4	222,63	0,6	0,22	14,19	12,2
1609,9	342,89	0,49	0,28	14,49	5,2
992,6	180,34	1,26	0,59	6,22	0
404,3	122,03	0,1	0,14	4,47	10,33
2010,9	31,22	4,62	7,83	10,04	33,28
699,8	211,03	1,18	0,5	5,36	0
2280	143,28	1,54	1,93	4,79	0
1123,7	182,22	1,17	0,24	4,83	5,77
480,8	217,75	0,57	0,37	13	17,41
693,3	73,59	5,76	0,89	12,97	12,75
564,7	148,16	0,21	0,17	6,04	11,5
645,7	238,11	0,03	0	5,81	0
1042,7	210,78	0,06	0,95	8,3	0
1520,7	236,56	0,09	0,17	5,7	4,56
1707,9	253,89	0,95	0,21	12,67	13,6
1232,8	469,16	0,29	0,07	8,03	21,05
547,9	192	0,35	0,52	11,49	16,33
1805,8	551,96	0,12	0,22	15,14	12,35
1920,4	208,04	0,36	0,05	14,61	12,57
726,8	288,57	0,22	0,2	11,88	9,13
290,8	284,48	0,03	0,15	7,14	5,04
6627,6	86,44	4,64	0,51	10,62	32,44
701,7	215,59	0,16	0,37	7,36	8,35
1372,5	362,6	1,32	0,22	10,96	33,65
494,8	180,05	0,06	0	2,64	3,91
1087,4	122,03	0,69	0,35	3,53	0
469,6	166,43	0,04	0,05	2,68	0

3102,8	189,71	2,46	0,97	14,79	5,14
2373,2	245,04	0,51	0,13	13,91	3,04
729,6	253,88	0,25	0,17	6,55	3,51
986,8	307,23	0,24	0,14	12,74	1,79
2585,7	317,26	1,05	0,03	14,28	15,82
3228,6	263,37	0,19	0,19	2,91	6
2513	91,61	3,41	0,96	2,09	32,09
799,5	165	0,63	1,07	3,09	3,12
1501,1	126,96	3,7	1,88	7,6	17,73
749,2	289,99	0,27	0,05	8,79	2,08
3491,3	99,59	4,67	2,53	17,68	12,48
2180,3	394,13	0,13	0	3,66	12,97
2146,8	145,63	1,56	1,14	14,11	1,04
3446,6	254,53	2,1	1,59	17,57	9,74
1668,8	205,66	0,18	0	10,99	2,09
1884	141,56	1,92	1,17	16,08	4,79
715,6	288,4	0,13	0,25	17,33	4,29
2090,9	194,26	1,65	0,92	17,16	3,81
2358,69	260,95	0,17	0,39	16	8,06
549,77	379,78	0,04	0,19	6,6	0
337,53	165,5	0,01	0	2,89	1,24
859,96	130,28	1,57	0,25	6,98	5,28
683,64	253,59	0,07	0,35	7,23	7,39
1493,41	142,82	1,86	0,82	9,38	3,16
2495,83	261,39	1,27	0,22	9,92	2,98
1630,55	229,79	0,71	0,16	3,23	0
3282,74	146,47	2,79	0,21	6,97	2,19
6283,46	133,32	3,85	3,93	16,88	26,75
3638,65	195,29	2,4	0,64	17,01	11,66
1450,97	349,77	0,28	0,13	7,52	3,84
2006,05	202,28	1,63	0,35	7,21	3,79
4379,85	175,16	2,93	0,82	10,47	15,13
4219,86	79,26	6,03	1,83	17,44	15,09
1826,46	323,44	0,39	0,16	8,27	3,56
1281,18	238,45	1,13	0,15	6,25	2,81
2835,41	248,29	2,1	0,1	11,91	7,64
2600,32	402,96	0,88	0,11	7,65	2,42
4846,77	259,04	1,2	0,32	12,67	30,54
1872,18	245,1	0,42	0,06	1,33	0
4902,28	461,42	1,15	0,26	6,7	0
3739,87	368,41	1,32	0,03	5,53	0
3984,76	217,16	2,04	0,26	8,08	0,62
7279,35	239,89	2,4	0,15	11,6	6,16
2982,34	606,17	0,17	0,16	13,87	5,99
582,42	137,75	0,87	0,89	4,28	1,31
1555,45	329,35	0,84	0,52	10,64	0,79
1565,25	219,3	0,49	0,18	5,24	1,32
2022,38	288,97	1,26	0,41	9,72	0,63
3063,97	118,2	2,32	1,35	16,18	7,62
311,41	265,24	0,15	0,2	6,67	1,04

1082	136,21	2,46	0,82	1,43	0
1245,26	117,8	1,98	2,2	2,83	0
1170,16	388,5	0,33	0,08	0,26	0
814,25	113,69	0,96	1,73	5,11	1,31
1339,95	136,95	1,93	0,44	12,57	1,83
1023,22	165,96	1,42	0,28	4,48	0
745,68	108,86	0,72	0,82	4,32	0
288,55	172,27	0,36	0,09	0,83	0
2188,9	441,33	0,95	0,19	1,72	0
1305,1	84,79	4,07	1,67	8,85	0
810,6	349,68	0,04	0,13	1,81	0
1903,3	131,4	2,56	1,21	10	0
1515,2	309,58	0,33	0,03	8,77	0
1105,7	217,96	0,32	0,23	3,85	0
1071,2	512,41	0,01	0,09	0,52	26,04
3320,5	23,87	18,51	11,44	0,56	26,83
690,9	209,46	0,47	0,39	0	0
709,6	125,17	1,48	0,57	0	0
584,6	166,56	0,14	0,54	0,03	0
2262,3	164,45	2,27	0,25	2,19	25,74
3955,9	378,39	0,75	0,05	1,07	0
348	204,79	0,06	0,26	0	0
422,4	195,72	0,01	0	0,4	0
762,7	319,47	0,03	0,08	3,15	0
387,8	154,83	0,03	0,17	0,78	0
1475,3	148,84	2,76	1,29	1,82	0
457	814,76	0,03	0	8,5	29,07
4397,3	324,5	1,76	0,8	11,8	26,83
446,3	254,75	0,1	0,05	7,28	4,32
4229,8	211,86	2,48	0,36	13,09	26,45
1251,9	147,27	0,83	0,59	9,32	5,51
1267,9	86,49	3,37	1,05	8,51	16,14
946,2	114,71	1	0,5	5,87	0,6
645,7	619,49	0,01	0	5,69	6,35
4524,9	182,34	2,89	0,56	7,87	22,89
576,6	109,63	0,19	0	1,26	1,02
1451,4	171,4	0,32	0,16	6,23	3,01
464,9	228	0,05	0,05	2,21	0
5327,9	24,08	24,61	9,31	4,27	13,39
1557,7	267,55	0,11	0,08	2,97	0,21
898,3	137,65	0,81	0,78	2,3	0
927,6	328,41	0,06	0,23	3,63	8,35
3384,3	286,86	1,1	0,28	3,46	7,65
507,5	184,55	0,09	0,06	0,36	7,56
3823	544,02	0,39	0,14	4,02	10,47
2267,6	149,44	2,09	0,96	3,62	3,06
2015	165,86	0,3	0,15	3,43	3,99
1220	443,8	0,06	0,02	1,41	0
1031,3	339,61	0,1	0,07	0,15	0
3469,4	203	2,03	0,1	3,3	13,91

1952,48	224,03	0,42	0,26	8,28	55,29
455,7	285,71	0,93	0,18	9,33	37,35
1400,1	139,06	1,72	0,62	7,62	16,66
4697,6	107,77	3,29	0,25	9,2	43,26
1376,3	340,97	0,75	0,04	8,07	24,72
2844,5	0	0	0	9,02	30,09
3864,3	303,01	1,33	0,09	8,97	41,63
2209,6	191,1	2,56	0,84	8,7	21,82
2078,7	151,34	1,18	0,26	4,98	7,8
979,5	248,19	0,37	0,43	7,22	6,37
852,5	119,88	1,86	0,72	7,76	18,94
558,9	94,06	1,85	0,93	8,49	21,42
983,5	136,08	2,26	1	8,86	11,02
3800,8	56,76	6,55	1,04	6,71	4,3
1658	618,14	0,33	0,03	7,42	14,24
769,2	141,24	0,33	0,54	5,45	0
931,9	142,69	1,94	0,66	6,49	14,53
201,7	201,59	0,2	0	9	22,53
598,6	72,35	0,96	0,87	8,77	23,94
5324,6	197,9	2,28	0,33	10,22	39,19
2177,9	93,82	2,77	0,64	9,25	3,97
0	85,93	0,75	0,24	4,55	15,76
19,2	379,05	0,06	0	6,91	16,89
947,7	187,82	1,87	0,68	10,21	22,78
1102,5	68,24	2,66	1,1	9,8	17,85
765,2	106,6	1,67	0,65	8,96	33,27
3046,9	81,13	5,06	2,4	10,42	27,77
769,2	366,98	0,46	0,13	6,91	14,94
761,2	198,71	0,76	0,13	6,24	5,43
658,1	280,08	1,5	0,01	2,6	49,34
3979,4	54,61	4,4	2,61	5,21	34,01
0	180,39	0,3	0,08	3,56	36,54
0	314,93	0,04	0,03	3,95	24,89
118,4	263,79	0,14	0,06	3,75	1,28
495,4	210,79	0,47	0,59	5,88	0,32
6550,7	26,12	19,54	2,66	9,46	16,18
0	201,47	0,14	0,13	6,67	10,3
269,2	162,82	0,18	0,1	6,21	7,21
0	49,14	0,72	1,55	3,85	2,82
1983,4	57,24	2,89	0,47	8,48	8,14
1427,9	256,87	1,33	0,24	6,32	8,52
3432,9	291,64	2,12	0,19	11,42	27,51
480,8	104,68	0,74	0,55	3,78	9,59
734,1	267,21	0,16	0,1	6,16	9,13
1095,8	92,42	3,8	1,06	8,77	16,1
1207,6	64,7	3,89	1,93	9,38	9,14
2317,3	146,31	2,2	0,88	11,17	13,74
963,6	138,31	0,69	0,89	7	8,42
1522,9	183,26	0,31	0,61	7,02	4,89
2514,8	211,52	2,51	1,27	12,32	19,34

562,6	84,15	0,47	0,46	7,16	22,43
1918,6	702	0,18	0,15	9,16	28,36
1055,9	80,09	3,65	1,18	10,66	15,31
1778,8	108,43	2,28	0,24	12,16	14,39
2024,1	164,57	1,09	0,25	13,33	11,89
647	156,49	0,32	0,22	8,6	5,98
565,3	240,14	0,16	0,21	10,97	4,87
5319,1	185,21	3,43	1,05	15,61	26,54
3287,8	95,53	3,56	0,53	11,58	15,45
1855,3	71,07	2,88	1,23	10,74	18,31
1343,5	92,95	1,48	1	13,79	9,12
504,6	123,23	0,31	0,29	9,22	4,9
567,9	150,13	0,57	0,58	12,44	8,11
1517,6	178,92	0,74	0,51	10,86	12,46
902,9	180,52	0,39	0,33	7,66	22,07
1016,4	266,17	0,61	0,29	9,15	32,08
1456,9	129,83	2,82	1,13	13,67	36,99
1773,5	224,2	0,82	0,29	11,32	35,84
1797,2	63,28	3,62	0,48	11,99	30,9
2216,7	181,8	2,01	0,07	10,08	2,14
3055,6	51,39	8,78	2,22	15,65	15,73
4904,9	25,06	20,43	7,57	14,28	13,21
1639	212,49	0,91	0,62	18,54	27,57
2564,9	147,46	2,73	0,41	13,83	14,01
3163,8	144,91	2,73	0,77	9,99	32,85
1079,7	95,99	3,81	1,25	9,08	7,88
2311,7	106,52	1,07	1,73	8,87	0
2124,4	140,86	4,14	0,92	10,18	30,4
1106,1	244,53	1,96	0,38	9,81	10,37
1063,9	132,77	4,31	0,87	9,12	26,84
3555,6	87,08	6,74	0,89	13,65	22,98
1246,7	225,14	0,67	0,08	10,66	12,54
989,6	96,59	0,27	0,48	8,45	12,84
665,3	353,84	0,08	0,23	13,75	19,21
1551,4	402,1	0,23	0,05	10,68	24,13
3463,4	81,62	2,44	1,45	14,17	26,91
2090,9	100,76	2,4	1,33	6,19	2,14
623,4	327,62	0,08	0,11	0,84	4,77
1397,7	175,09	1,59	0,59	16,37	6,12
1302,6	215,26	0,42	0,32	10,17	6,17
1431,2	152,81	0,74	0,83	12,63	15,97
1011,9	154,21	1,13	0,32	10,39	9,06
2135,6	310,96	0,29	0,29	12,74	12,06
1003,5	163,14	0,41	0,67	11,66	16,57
1453,6	173,8	0,29	0,32	6,45	29,4
522,8	200,34	0,07	0,07	6,36	21,76
2015,4	262,03	0,78	0,53	9,19	18,38
2350,9	285,93	0,91	0	11,42	21,93
2996,6	209,79	1,23	0,43	14,58	7,24
3471,7	130,36	2,8	0,83	9,34	8,72

698,9	435,63	0,05	0,07	5,05	0,3
167,8	337,31	0,07	0,01	4,29	2,87
2334,1	629,58	0,58	0,16	9,02	3,27
2247,4	362,35	0,79	0,5	13,76	4,68
4701,7	355,09	1,29	0,03	3,4	3,43
525,6	199,38	0,42	0	0,69	0
936,5	170,17	0,12	0,4	2,31	0
2532,5	103,5	1,51	2,1	10,39	2,21
3153,1	403,35	0,9	0,25	10,96	2,61
2381,6	203,73	0,51	0,38	11,94	1,97
2417,9	279,87	1,64	1,33	12,3	5,03
1752,7	331,21	0,55	0,15	16,12	1,57
2185,9	181,54	1,86	0,48	4,28	12,33
7086	146,67	3,85	0,65	13,98	4,67
1610,1	217,94	1,24	0,35	4,41	0
5475,9	148,96	0,99	0,67	14,08	4,83
1011,9	255,66	0,07	0,07	8,86	0,2
1475,9	310,54	1,03	0,29	4,1	0
964,5	257,22	0,76	0,29	9,17	0,9
944,9	457,46	0,12	0,17	12,89	4,54
37,1	221,96	0,04	0	3,95	4,54
1346,5	273	0,49	0,31	12,88	8
2397,9	119,64	3,93	1,4	7,59	9,59
908,9	390,04	0,3	0,18	14,3	23,03
3153,1	396,11	0,84	0,01	8,32	16,1
6577,3	422,86	1,09	0,51	11,88	12,52
3733,3	132,79	3,49	3,42	17,25	29,83
1225,7	529,75	0,16	0,06	5,74	15,84
804,5	549,09	0,02	0,06	7	0,69
624,9	385,9	0,04	0	4,89	3,8
2727,7	253,09	0,93	0,06	5,14	0
2009,3	411,44	0,86	0,36	8,65	6,66
2636,2	417,64	0,8	0,19	19,43	18,41
6590,4	542,91	0,88	0,14	10,45	7,66
5963,5	41,7	11,41	1,24	11,67	5,18
3769,3	95,79	4,61	0,2	2,69	1,53
1023,2	266,65	1,46	0,52	2,88	11,58
3321,9	426,04	1,09	0,34	14,06	10
2616,6	322,18	0,37	0,25	6,43	3,58
5561,9	165,06	3,04	0,34	5,4	4,82
1979,9	395,7	0,62	0,11	4,63	5,79
1532,6	473,25	0,89	0,19	8,55	1,78
2169,3	262,11	1,56	0,25	2,13	3,76
1483,6	609,43	0,27	0,12	10,84	3,71
1016,7	342,25	0,06	0,25	3,25	0
1023,2	319,05	0,55	0,31	7,23	1,96
1016,7	246,65	1,23	0,19	1,7	0
739,2	383,91	0,43	0,39	2,83	0
2241,1	85,43	3,75	1,83	13,44	4,74
1137,5	262,78	1,37	0,3	10,1	5,33

76,3	104,54	0,05	0,05	5,53	0
651	311,03	0,69	0,17	7,12	2,09
2019,1	180,25	1,17	0,84	13,85	3,55
1268,1	140,06	1,04	0,53	3,42	0
1101,6	145,88	0,45	0,23	2,14	0
1526,1	152,85	0,84	0,23	4,22	0
2815,8	196,09	1,47	0	3,56	7,13
3139,1	268,58	1,82	0	10,18	4,24
4921,9	166,04	4,65	0,71	3,14	0
823,9	407,34	0,09	0,34	10,49	0
1427,4	128,25	0,36	0,9	7,44	0
1156,2	294,84	0,97	0,1	3,79	0
1289,2	109,58	1,36	0,67	2,1	0
1095,1	262,09	0,24	0,4	8,53	0
1065,8	469,99	0,25	0,2	0,66	70,16
1778,4	485,11	0,35	0,13	0	100
1733,2	343,18	1,46	0,04	0	100
1746,5	116,53	2,82	1,21	0,26	29,91
1140,3	334,95	0,13	0,32	0,15	0
895,7	231,31	0,08	0,62	5,83	10,8
760,1	266,95	0,32	0,27	4,31	1,24
760,1	255,88	0,08	0,16	2,88	6,43
2504,2	316,16	0,56	0,74	1,99	35,81
1512,5	251,27	0,18	0,58	1,61	29,65
688,3	180,23	0,03	0,15	2,79	1,48
3703,3	239,17	2,05	0,17	11,71	28,3
869,1	237,74	0,02	0,23	9,03	7,47
2068,2	433	0,68	0,27	12,35	21,59
1382,2	173,39	0,63	0,06	5,97	11,22
1613,5	322,44	1,07	0,21	8,02	24,09
2150,6	257,93	1,76	0,81	9,18	24,12
2331,4	247,83	0,61	0,71	5,83	10,37
2775,4	159,63	1,47	0,55	7,76	8,27
3067,9	117,7	3,17	0,34	4,55	21,26
7311,3	325,19	1,63	0,03	1,11	0
2363,3	362,2	0,86	0,01	3,68	0
1799,7	230,19	1,47	0,54	6,24	0,31
624,5	197,64	0,49	0,19	1,81	9,93
1666,7	285,76	1,13	0,02	1,03	19,03
512,8	272,08	0,04	0	0,23	1,8
2262,3	174,28	2,16	1,11	3,57	8,53
1065,8	318,27	0,15	0,01	2,04	5,91
730,8	196,75	0,06	0	0,99	0
2387,2	236,83	1,96	0,03	2,51	0
1438,1	344,26	0,05	0,06	0,36	0
3299,2	154,67	3,18	0,41	0,56	32,24
3251,4	290,08	1,47	0,06	3,46	20,77
3870,8	55,97	9,16	5,19	4,55	21,26
2554,8	294,76	1,15	0,18	3,95	0,94
4075,6	468,27	1,14	0,02	6,98	28,83

1537,76	267,6	0,75	0,75	6,22	6,35
